# Age, gender, will, and use of home-visit nursing care are critical factors in home care for malignant diseases; a retrospective study involving 346 patients in Japan

**DOI:** 10.1186/1472-684X-10-17

**Published:** 2011-11-01

**Authors:** Yuko Kodama, Tomoko Matsumura, Takuhiro Yamaguchi, Morihito Takita, Shohei Kawagoe, Yukihiro Kimura, Satoshi Hirahara, Hiroshi Suzuki, Hideki Ohta, Shigeru Onozawa, Tadashi Wada, Yukiyasu Nakamura, Kazushi Nakano, Masahiro Kami, Koichiro Yuji

**Affiliations:** 1 Division of Social Communication System for Advanced Clinical Research, the Institute of Medical Science, the University of Tokyo, Tokyo, Japan; 2 Clinical Data Management Division, The University of Tokyo, Tokyo, Japan; 3 Aozora Medical Clinic, Chiba, Japan; 4 Morioka Home Care Clinic, Iwate, Japan; 5 Kajiwara Clinic, Tokyo, Japan; 6 Suzuki Clinic, Tokyo, Japan; 7 Oyama -johoku Clinic, Tochigi, Japan; 8 Departments of Family Medicine, Kameda Medical Center, Chiba, Japan; 9 Hinode Clinic, Fukuoka, Japan; 10 Nakano Home Care Clinic, Kagoshima, Japan; 11 Department of Internal Medicine, the Institute of Medical Science, the University of Tokyo, Tokyo, Japan

**Keywords:** palliative medicine, cancer, dementia, complication, performance status

## Abstract

**Background:**

We aimed to clarify the factors affecting outcomes of home care for patients with malignant diseases.

**Methods:**

Of 607 patients who were treated in 10 clinics specialized in home care between January and December 2007 at Chiba, Fukuoka, Iwate, Kagoshima, Tochigi and Tokyo prefectures across Japan, 346 (57%; 145 men and 201 women) had malignant diseases. We collected information on medical and social backgrounds, details of home care, and its outcomes based on their medical records.

**Results:**

Median age of the patients was 77 years (range, 11-102), and 335 patients were economically self-sufficient. Their general condition was poor; advanced cancer (n = 308), performance status of 3-4 (n = 261), and dementia (n = 121). At the beginning of home care, 143 patients and 174 family members expressed their wish to die at home. All the patients received supportive treatments including fluid replacement and oxygenation. Median duration of home care was 47 days (range, 0-2,712). 224 patients died at home. For the remaining 122, home care was terminated due to complications (n = 109), change of attending physicians (n = 8), and others (n = 5). The factors which inhibited the continuity of home care were the non-use of home-visit nursing care (hazard ratio [HR] = 1.78, 95% confidence interval [CI]: 1.05-3.00, *p *= 0.03), the fact that the patients themselves do not wish to die at home (HR = 1.83, CI: 1.09-3.07, *p *= 0.02), women (HR = 1.81, CI: 1.11-2.94, *p *= 0.02), and age (HR = 0.98, CI: 0.97-1.00, *p *= 0.02).

**Conclusions:**

Continuation of home care is influenced by patients' age, gender, will, and use of home-visit nursing.

## Background

Most cancer patients prefer to die at home [1]. In response to the wishes of such patients, home care for terminally ill cancer patients has been developed since the 1990s, and studies on this issue are still increasing in number [2]. In home care, patients can be treated while taking into consideration the set of values and lifestyle habits of each individual.

Home care has provided a new treatment option for cancer patients, while it remains unknown who are optimal candidates for this treatment. Several researchers reported that success of home care is influenced by medical conditions, incomes, and education levels of patients and their families [3-5]. These studies provided us valuable information on home care; however, they are small-sized studies with their backgrounds varied widely. The factors influencing performance of home care have not yet been elucidated.

To investigate the factors that influence continuation of home care for patients with malignant diseases, we conducted a retrospective study involving 364 cancer patients who were treated at home.

## Methods

### Data collection

This study was conducted with the participation of 10 clinics specialized in home care at Chiba, Fukuoka, Iwate, Kagoshima, Tochigi and Tokyo prefectures across Japan. Oncologists worked as full-time staffs in all the clinics.

Patients who have been mainly affected by cancer were selected from 607 patients who received home care between January 1^st ^and December 31^st ^2007. We collected information on medical and social backgrounds, details of home care, and its outcomes using their medical records. The parts of information that could not be assessed from medical records were investigated by asking the attending physicians. Performance status (PS) was classified according to the criteria of the Eastern Cooperative Oncology Group [6].

### Endpoints and statistical analysis

This study aimed to elucidate the factors influencing the continuation of home care for patients with malignant diseases. Data were analyzed as of December 31^st ^2007.

The cumulative incidences of death at home and hospitals were evaluated using the Gray's method [7]. Associations between potential prognostic factors and outcomes were evaluated using the Cox's proportional hazard regression models. Events were defined as discontinuation of home care due to causes other than deaths at home. Data on patients who died at home were censored. The following variables were considered: patients' age at the beginning of home care, gender, presence of dementia, presence of patients' wish to die at home at the beginning of home care, residence (patients' own home or a facility), and the concomitant use of home-visit nursing care. Variables that had a *p *value of less than 0.25 on univariate analysis were entered into the mixed-effects model. Those that contributed less than 10 percent to the overall ability of the model were sequentially eliminated. The level of significance was set at *p *< 0.05.

For the implementation of this study, we have obtained approval of the Ethical Review Board of the Institute of Medical Science, the University of Tokyo (IRB approval number 19-7).

## Results

### Medical backgrounds

Of the 607 patients who received home care at the 10 clinics, 346 (57%) had malignant diseases. Medical backgrounds of the patients were shown in Table 1. Most patients were in poor general conditions, and had several complications other than malignant diseases.

**Table 1:** Patients' medical backgrounds.

Variables	n
Age	
< 35 y	3
35-50 y	9
51-60 y	33
61-70 y	69
71-80 y	108
81-90 y	86
> 90 y	38
Gender	
Men/women	145/201
Performance status*	
1/2/3/4/unknown	40/27/153/108/18
Types of malignant diseases	
Lung cancer	80
Gastric cancer	47
Pancreatic cancer	17
Prostate cancer	16
Rectal cancer	16
Hematological malignancy	14
Cholangiocarcinoma	13
Liver cancer	12
Colorectal cancer	11
Breast cancer	10
Uterine cancer	10
Bladder cancer	9
Renal cancer	9
Ovarian cancer	8
Esophageal cancer	6
Gallbladder cancer	5
Laryngeal cancer	5
Renal pelvis cancer	5
Cancers of unknown origin	6
Others	47
Complications	
Dementia	121
Cerebrovascular disease	24
Metastasis	135
Patients with two or more cancers	38
Cancer pain	81
Paralysis	48

*1: Performance status according to the Eastern Cooperative Oncology Group

### Social backgrounds

Social backgrounds of the patients were shown in Table 2. Of the 346 patients, 335 (97%) were economically independent, and 336 (97%) lived with their families or others.

**Table 2:** Social backgrounds of the patients and their families.

Variables	n
Household type	
Solitary	10
Elderly couple	32
Living with family, but solitary in daytime or nighttime	59
Others*	245
Residence	
Own house	332
Others	14
Economic conditions	
Independent	335
Receiving public assistance	8
Economic difficulties	3

* Some family members or caregivers lived together throughout the day.

### Treatment at home

At the beginning of home care, neither the patients themselves nor their families wished to receive any aggressive cancer treatment. All the patients received supportive treatments on the basis of the administration of fluid replacement and oxygenation. Three received intravenous hyperalimentation. Of 81 patients who complained of cancer pain, 74 were given narcotic medications. Their adverse effects were controllable in these 74 patients.

Eleven patients received red cell transfusion at home. There were no particular complications. No patients received platelet transfusion.

### Patients' and their family members' wish to die at home

At the beginning of home care, 143 patients (41%) and 174 family members (50%) expressed their wish to die at home. Of the 143 patients, 122 (85%) were in agreement with their families' wishes. Right before death, 145 (41%) patients and 213 (62%) families expressed their wish to die at home.

Four patients and seven families withdrew their wish to die at home in the midcourse of home care. For the patients, the reasons were an increased burden on the family or caregivers (n = 3), and living alone (n = 1). Meanwhile, for the family members, the reasons were fear for sudden changes in the patients' conditions (n = 5) and an increased burden on the family (n = 2).

### Outcomes

**Figure 1:**
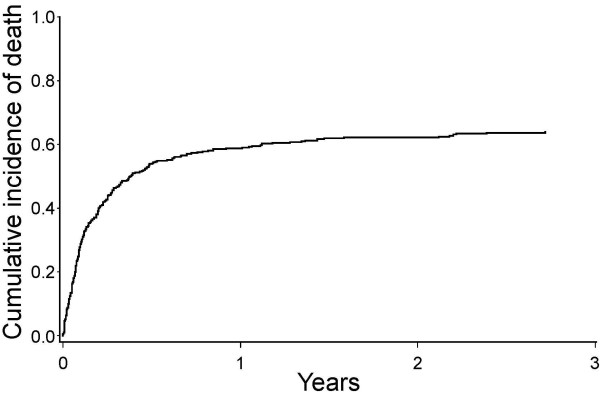
**Cumulative incidence of deaths during home care**. X axis indicates duration of home care (years).

The median duration of the home care was 47 days (range, 0-2,712). The cumulative mortality during the home care was shown in Figure 1.

224 patients died at home. For the remaining 122, home care was terminated for reasons other than death. They included hospitalization due to the complications (n = 109), change of attending physicians (n = 8), admission to a nursing home (n = 3), return to outpatient treatment after improvement of the disease condition (n = 1), and disappearance (n = 1).

13 patients developed acute complications. 11 of them were hospitalized after ambulance transportation, and the home care was terminated. The two remaining patients died at home.

### Factors inhibiting the continuitation of home care

The results of a multivariate analysis were shown in Table 3. The factors which inhibited the continuation of home care were the non-use of home-visit nursing care (hazard ratio [HR] = 1.78, 95% confidence interval [CI]: 1.05-3.00, *p *= 0.03), the fact that the patients themselves do not wish to die at home (HR = 1.83, CI: 1.09-.3.07, *p *= 0.02), women (HR = 1.81, CI: 1.11-2.94, *p *= 0.02), and age (HR = 0.98, CI: 0.97-1.00, *p *= 0.02).

**Table 3:** Multivariate analysis on factors inhibiting the continuity of home care

Factors	Tested variables	Hazard ratio	95% confidence interval	*p *value
Age	Increasing age	0.98	0.97-1.00	0.02
Gender	Women	1.81	1.11-2.94	0.02
Dementia	Present	0.64	0.38-1.09	NS
Patient's wish to die at home at the beginning of home care	No wish	1.83	1.09-3.07	0.02
Residence	Nursing home	2.98	0.86-10.25	NS
Visiting Nurse	No use	1.78	1.05-3.00	0.03

NS: not significant.

## Discussion

This study revealed that medical backgrounds of cancer patients receiving home care in Japan showed common clinical features. For example, 322 (93%) of the patients had solid cancers, and the majority of the cancer types were lung (n = 80, 23%) and gastric cancer (n = 47, 14%). Our study reflects the situation in Japan, in a similar way as previous studies conducted in other countries [8]. Lung cancer is the primary cause of cancer death, and gastric cancer is in the second place in Japan [9]. Meanwhile, patients with hematologic malignancies were rarely treated at home. The occurrence of sudden changes due to complications and need for adjuvant therapy such as blood transfusion and palliative chemotherapy were probably the obstacles for these patients [10].

Most patients were elderly, and had several complications. For example, 232 (67%) of the patients were 71 years old or older, 261 (75%) were PS 3-4, and 121 (34%) were complicated with dementia. The subjects in this study were older than those in previous studies reported from other countries [11,12]. These findings suggest that even severely ill patients with poor general conditions can be treated at home by providing adequate care.

The therapeutic interventions conducted during home care were mainly symptomatic treatments. In fact, all the patients received supportive medications. Pain control is a major aim in patients with advanced cancer [13]. In this study, 81 patients (23%) complained intense pains. Among them, 74 were given narcotic medications without serious complications. These findings were comparable to previous studies [12]. Since most physicians who provide home care are familiar with palliative medicine, palliative treatment using narcotics can be provided safely at home.

The majority of cancer patients who received home care was financially independent, and did not receive any public financial assistance. Such financial situations were similar to those of previous studies [14,15]. Home care might be an option limited to patients with sufficient financial resources since it can increase the economic burden on the patients. Meanwhile, 336 (97%) received support from their families or from the staff of the facility. Even patients who had lived alone and died at home received nursing care by their families during their last moments, suggesting that family or social supports are requisite for cancer patients to live their last moments at their own homes.

This study showed that use of home-visit nursing care services, will of the patients, their gender and age played an important role in the continuation of home care. It is reasonable to speculate that the use of home-visit nursing care services contributes to the safe in home care since medical providers visit more frequently and patients are closely monitored. Women were found as a significant factor for inhibiting the continuitation of home care in this study while a previous study from Taiwan showed that being female was an important determinant of hospice home care [16]. In a meta-analysis using studies mostly from the UK, the US, Australia, and Canada, gender factor did not have significant effect in dying at home [17]. Thus, differences of culture among countries might influence the gender disparity. In Japan, elderly care has been traditionally considered as 'women's work' [18] and national survey reported that 69.4% of caregivers were women in 2010 [19]. An explanation of the gender disparity might be that Japanese women miss the opportunity to receive medical home care in the background of traditional thinking of gender role. Surprisingly, older age was associated with longer continuation of home care. The exact reason remains unknown; however, it is probable that cancer might have been diagnosed by chance at an early stage in elderly patients who had been treated at home for other chronic diseases.

This study provided valuable information on home care for cancer patients; however, it still involves several problematic issues. The most considerable problem is that this was a small-scale retrospective study. For that reason, it might be accompanied by a bias of which researchers were unaware. For example, because this study was conducted with physicians with a rich experience in home care, caution is required when generalizing these results. To overcome these problems, validation by large-scale prospective studies should be performed in the future.

## Conclusions

This study revealed common clinical feature of cancer home care in Japan: the majority of the population received home care was elderly (67% of patients over 70 years of age), had poor performance status (75% patients with PS 3-4) and had dementia (34%). Regarding social background, 97% of cancer patients were economically independent and lived with their families or others. Age, gender, will and use of home-visit nursing care had significant impact on the continuation of home care. A large, international cohort study to investigate the continuation of cancer home care should be considered since this study has limitations such as nature of small sample size and domestic study in Japan.

## Competing interests

The authors declare that they have no competing interests.

## Authors' contributions

Contribution: YKo and SK designed the research and wrote the paper. YKo, TM and TY analyzed data. YKo. SK and MT performed research. YKi, SH, HS, HO, SO, TW, YN, KN, and KY managed the data. SK took the leadership of authors. All authors read and approved the final manuscript.

## Pre-publication history

The pre-publication history for this paper can be accessed here:

http://www.biomedcentral.com/1472-684X/10/17/prepub
